# Loxapine to control agitation during weaning from mechanical ventilation

**DOI:** 10.1186/s13054-017-1822-y

**Published:** 2017-09-06

**Authors:** Stéphane Gaudry, Benjamin Sztrymf, Romain Sonneville, Bruno Megarbane, Guillaume Van Der Meersch, Dominique Vodovar, Yves Cohen, Jean-Damien Ricard, David Hajage, Laurence Salomon, Didier Dreyfuss

**Affiliations:** 1 Service de Réanimation Médico-Chirurgicale, Hôpital Louis Mourier, Assistance Publique – Hôpitaux de Paris, F-92700 Colombes, France; 2 Epidémiologie Clinique et Évaluation Économique Appliqué aux Populations Vulnérables (ECEVE), UMR 1123, Université Paris Diderot, Sorbonne Paris Cité, F-75018 Paris, France; 3 Service de Réanimation Polyvalente et surveillance continue, Hôpital Antoine Béclère, Assistance Publique – Hôpitaux de Paris, F-92140 Clamart, France; 4 INSERM U999, Université Paris Sud, F-92060 Le Plessis Robinson, France; 5 Service de Réanimation Médicale et maladies infectieuses, Hôpital Bichat, Assistance Publique – Hôpitaux de Paris, Paris, France; 6 Service de Réanimation Médicale, Hôpital Lariboisière, Assistance Publique – Hôpitaux de Paris, Paris, France; 7 Service de Réanimation Médico-Chirurgicale, Hôpital Avicenne, Assistance Publique – Hôpitaux de Paris, Bobigny, France; 8 Infection, Antimicrobials, Modelling, Evolution (IAME), UMR 1137, Université Paris Diderot, Sorbonne Paris Cité, F-75018 Paris, France; 9 Département d’Epidémiologie et Recherche Clinique, Hôpital Louis Mourier, Assistance Publique – Hôpitaux de Paris, 178 rue des Renouillers, F-92700 Colombes, France; 10 Present address: Intensive Care Unit, Hôpital Louis Mourier, 178 rue des Renouillers, 92110 Colombes, France

**Keywords:** Mechanical ventilation, Ventilation weaning, Agitation, Intensive care unit

## Abstract

**Background:**

Weaning from mechanical ventilation (MV) may be impeded by the occurrence of agitation. Loxapine has the ability to control agitation without affecting spontaneous ventilation. The aim of this study was to establish whether loxapine would reduce MV weaning duration in agitated patients.

**Methods:**

We performed a multicentre, double-blind, placebo-controlled, parallel group, randomised trial. Patients who were potential candidates for weaning but exhibited agitation (Richmond Agitation-Sedation Scale score ≥ 2) after sedation withdrawal were randomly assigned to receive either loxapine or placebo. In case of severe agitation, conventional sedation was immediately resumed. The primary endpoint was the time between first administration of loxapine or placebo and successful extubation.

**Results:**

The trial was discontinued after 102 patients were enrolled because of an insufficient inclusion rate. Median times to successful extubation were 3.2 days in the loxapine group and 5 days in the placebo group (relative risk 1.2, 95% CI 0.75–1.88, *p* = 0.45). During the first 24 h, sedation was more frequently resumed in the placebo group (44% vs 17%, *p* = 0.01).

**Conclusions:**

In this prematurely stopped trial, loxapine did not significantly shorten weaning from MV. However, loxapine reduced the need for resuming sedation.

**Trial registration:**

Clinicaltrials.gov, NCT01193816﻿. Registered on 26 August 2010.

## Background

Agitation is a frequent complication of critical illness, occurring in up to 50% of mechanically ventilated patients in the medical-surgical intensive care unit (ICU) [1]. It is associated with severe adverse events, including increased odds of unplanned extubation and central venous catheter removal, more frequent nosocomial infections and an increased duration of ICU stay [1]. Some preventive measures, including systematic evaluation of pain and agitation, have proven beneficial in reducing the incidence of agitation in the ICU [2]. Other measures, such as the use of sedation protocols to hasten weaning from mechanical ventilation (MV), may be associated with an increase in the incidence of agitation episodes [3, 4]. Current guidelines do not recommend the use of haloperidol or other typical antipsychotics to treat delirium and/or agitation [5]. Moreover, data on the safety of antipsychotics in the ICU setting and their efficacy to control agitation and improve patient outcomes are scarce. A randomised, double-blind, placebo-controlled trial of intravenous haloperidol in critically ill patients showed no effect on delirium incidence or duration. A secondary data analysis showed that the proportion of patients with a Richmond Agitation-Sedation Scale (RASS) score ≥ 2 was lower in the haloperidol group than in the placebo group (13% vs 20%, *p* = 0.0075), suggesting that it could be used for short-term management of acute agitation, despite having no effect on delirium [6].

Loxapine is a typical antipsychotic drug. A plasmatic peak is reached roughly 1.5 h after oral intake, and its half-life is around 8 h. Side effects include drowsiness, extrapyramidal symptoms, tachycardia and hypotension. Its approved indications in France are acute and/or chronic psychotic states and severe agitation. It has been used for a long time, mainly in France, because of its efficacy, safety profile, availability and cost. During a physiological study, loxapine was found to be safe and effective in treating agitation in mechanically ventilated patients and accounted for improved respiratory parameters [7]. In this study, we aimed to test whether the use of loxapine in agitated patients during weaning from MV could reduce the duration of MV.

## Methods

### Study design

This prospective, randomised, double-blind, multicentre study was conducted over a 2-year period (November 2011 to November 2013) in five university-affiliated French ICUs. Written informed consent was obtained from patients’ next of kin at inclusion in the study. In the absence of next of kin, informed consent could be waived, as allowed by the institutional review board according to French law that allows inclusion of patients in emergency conditions. Whatever the type of inclusion, all surviving patients were informed about the trial at the earliest opportunity after neurological recovery, and consent to continue in the trial was sought. An investigator at each centre was responsible for enrolling patients in the trial, following the protocol and completing the case report form. Two clinical research technicians regularly monitored the data.

The protocol was approved by an institutional review board, the Comité de Protection des Personnes Paris Ile de France I, according to French law. The study received no commercial support. This study was granted permission by the French Ministry of Health (PHRC P070106) and registered with ClinicalTrials.gov under the identifier NCT01193816.

### Participants

Adult patients under MV for > 48 h, meeting readiness-to-wean criteria (*see below*) and exhibiting agitation, as defined by a RASS score ≥ 2 [8], at the time of sedation decrease or withdrawal were eligible and included in the absence of non-inclusion criteria. Non-inclusion criteria included pregnancy, contra-indication to loxapine (e.g., hypersensitivity or known history of epilepsy), on-going treatment with a dopamine agonist, limitation of life-sustaining therapy, planned extubation in the following 24 h, and contra-indication to enteral access through a nasogastric tube.

### Randomisation and masking

Patients were randomised to receive loxapine or placebo immediately after inclusion. Study drug packs were prepared by the Louis Mourier Hospital Pharmacy Department (Colombes, France). The loxapine and placebo drug components of solution were packaged identically into numbered treatment packs matching the patients’ study numbers. Each treatment pack contained 14 flasks of 60 ml. Each flask contained either 1.5 g of loxapine or placebo used for the scheduled administrations over a 24-h period. Care providers and investigators were blinded to group assignment.

### Sedation management

In all participating centres, sedation consisted of continuous infusion of sufentanil and midazolam or propofol. Three centres used a written, nurse-implemented sedation protocol to reach an estimated appropriate depth of sedation on the basis of the RASS score. No written sedation protocol was used in the two other centres, and daily interruption of sedation was usual practice.

### Mechanical ventilation weaning

Readiness to wean was assessed in daily screening. The criteria were defined according to usual guidelines [9]: (1) recovery from acute disease (e.g., pneumonia, chronic obstructive pulmonary disease exacerbation, extra-pulmonary infection, shock); (2) marked improvement of the reason for MV initiation; (3) haemodynamic stability without catecholamine infusion or a small and decreasing infusion rate; and (4) peripheral capillary oxygen saturation ≥ 92%, fraction of inspired oxygen ≤ 50%, positive end-expiratory pressure ≤ 5 cmH_2_O and no sign of respiratory failure. Standard weaning from MV procedure was performed in all investigating centres [10]. The weaning test consisted of a T-tube trial with supplemental oxygen administration at four centres, and a pressure support trial with an inspiratory pressure of 5 cmH_2_O at one centre. The tube cuff was systematically kept inflated during T-tube trials.

Failure criteria encompassed respiratory rate > 35/minute, arterial oxygen saturation < 90%, heart rate > 140 beats per minute or sustained increase or decrease in heart rate > 20%, systolic blood pressure > 180 mmHg or < 90 mmHg, agitation, diaphoresis or anxiety. Arterial blood gas analyses could be performed but were not mandatory.

In case of weaning trial success, extubation was performed if physiotherapy evaluation revealed appropriate cough and muscular strength. Arterial blood gas analyses were not systematically performed. If respiratory acidosis (hypercapnia > 45 mmHg and pH < 7.35) was documented, the extubation could be delayed.

After extubation, patients received oxygen by mask or nasal cannula. Prophylactic non-invasive MV was not considered an exclusion criterion, because it can facilitate extubation of patients with chronic cardiac or respiratory impairment [11].

### Procedures

**Fig. 1:**
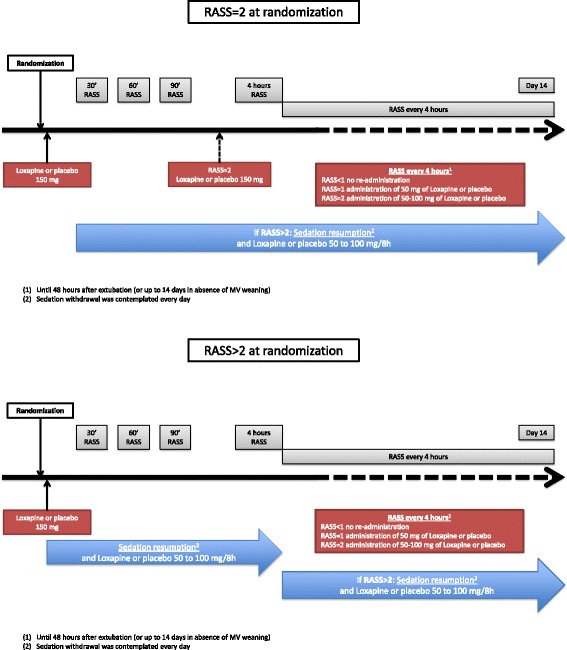
Drug protocol administration. *RASS* Richmond Agitation-Sedation Scale

During the study, no administration of a neuroleptic other than the study drug was allowed (Fig. 1).

#### In case of RASS score of 2 at randomisation

After randomisation, patients received 150 mg of loxapine or placebo by nasogastric tube. Our group has previously found a time of 62 ± 39 minutes between loxapine enteral administration and effect [7]. Therefore, to avoid agitation-related side effects such as unplanned tube or vascular line removal, patients received at the same time a short administration of a low dose of benzodiazepine and/or morphinic agents in order to give time for the effect of loxapine. RASS score was monitored after 30, 60 and 90 minutes as well as 4 h. A second dose of loxapine or placebo was administered between 90 minutes and 4 h after the first one in case of a persistent RASS score of 2.

Then, RASS score was monitored every 4 h until 48 h after extubation (or up to 14 days in the absence of MV weaning). This monitoring allowed us to adapt study treatment as follows: RASS score < 1, no loxapine or placebo re-administration; RASS score of 1, administration of 50 mg of loxapine or placebo; and RASS score of 2, administration of 50–100 mg of loxapine or placebo (at the discretion of the attending intensivist).

If the RASS score was found to be > 2 after randomisation, usual sedation (benzodiazepine and morphinic agents) was immediately resumed. Thereafter, 100 mg of loxapine or placebo was administered every 8 h, and sedation withdrawal was contemplated every day. When this allowed sedation withdrawal, loxapine administration was continued according to the modalities explained above (depending on RASS score).

#### In case of RASS score > 2 at randomisation

After randomisation, patients received 150 mg of loxapine or placebo by nasogastric tube, and usual sedation (benzodiazepine and morphinic agents) was resumed at the same time. Then, 100 mg of loxapine or placebo was administered every 8 h, and sedation withdrawal was contemplated every day. When this allowed sedation withdrawal, loxapine administration was continued according to the modalities explained above (depending on RASS score).

Within 48 h following extubation, administration of loxapine or placebo was pursued, depending on RASS score: no administration when the RASS score was < 1; administration of 0–50 mg (at the discretion of the intensivist) when the RASS score was 1; and administration of 50–100 mg (at the discretion of the attending intensivist) when the RASS score was 2. In case of RASS score > 2, control of agitation could include administration of a non-neuroleptic agent according to unit procedure.

### Data collection

At the time of inclusion, we recorded data on age, sex, Simplified Acute Physiology Score II (SAPS II) [12], indication for MV, co-morbid conditions, alcohol consumption, toxic drug abuse and psychoactive drug use. Amounts of sedative agents given in the previous 24 h were registered. The Sepsis-related Organ Failure assessment score [13] was recorded at the time of randomisation. Both RASS score and the following physiological variables were monitored at 30, 60 and 90 minutes as well as 4 h after inclusion: temperature, respiratory rate, heart rate, systolic arterial pressure and airway occlusion pressure during the first 0.1 second of inspiration (P0.1). Then, RASS score, need for repeat loxapine or placebo administration and sedation resumption were recorded every 4 h until 48 h after extubation or until day 14. Indication for sedation resumption, sedation duration and nosocomial pneumonia occurrence were also recorded. Vital status was monitored 6 weeks after randomisation. The patients were closely screened for the following loxapine side effects: dyskinesia, seizure and unexplained fever.

### Outcome

#### Primary outcome

The primary endpoint was the time between inclusion and successful extubation, as defined by the absence of re-intubation within 48 h after extubation.

#### Secondary outcomes

Secondary outcomes were the rate of unplanned extubation, ventilator-free days during the first 14 days, evolution of physiological parameters (in the first 4 h after randomisation), rate of sedation resumption in the first 24 h (this outcome has not been pre-specified on ClinicalTrials.gov), rate of nosocomial pneumonia, rate of loxapine side effects, mortality at 14 days and 6 weeks, and factors associated with unsuccessful weaning.

### Statistical analysis

Sample size was determined on the basis of previously reported data [10, 14] indicating that median duration of weaning from MV ranges between 3 and 9 days. With a median duration of 6 days in the placebo group and a follow-up period of 14 days, 115 patients per group were required to demonstrate a decrease of 2 days of median duration of MV (primary endpoint) in the loxapine group, with an alpha risk of 5% and a power of 80%. To take into account a death rate before successful extubation of about 25%, 150 patients per group were planned (300 patients overall).

All analyses were based on the intention-to-treat principle. Baseline characteristics were described by group (placebo, loxapine) using frequency and percent for categorical variables and mean and SD for continuous variables (except for continuous variables with a non-normal distribution, for which we used median and IQR). The primary outcome was represented using the Kaplan-Meier method. Time to successful extubation was calculated from the time of inclusion of the patient. Deaths before any successful extubation were censored at the time of death. Patients were censored after 14 days if no event (extubation or death) occurred during the follow-up. The primary outcome was compared between the two groups with a log-rank test.

For secondary endpoints, the chi-square test or Fisher’s exact test (as appropriate) was used to compare categorical variables, and Student’s *t* test or the Wilcoxon test (as appropriate) was used for continuous variables. Evolution of physiological (arterial pressure, heart rate, respiratory rate and temperature) and clinical (RASS score ≥ 2 or < 2) parameters during the first 4 h were compared between the placebo and loxapine groups with generalised estimating equation models in order to take repeated measurement of participants’ responses into account, and thus the expected correlation within each participant. An identity link function was used for continuous parameters, and a logit link function was used for binary parameters. An unstructured correlation structure was used in all models.

### Role of the funding source

The sponsor of the study (French Ministry of Health) had no role in study design, data collection, data analysis, data interpretation or writing of the report. SG, DH and DD had access to the raw data. The corresponding author (DD) had full access to all data and final responsibility for the decision to submit the report for publication.

## Results

### Baseline characteristics

**Fig. 2:**
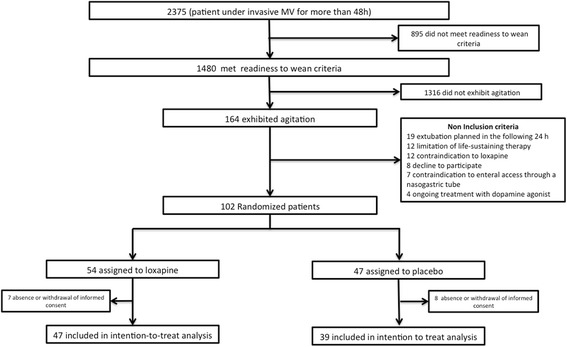
Patients flowchart of the 2-year study period

From November 2011 through November 2013, a total of 1480 patients under MV for more than 48 h and meeting readiness-to-wean criteria were present in the participating ICUs (Fig. 2). The trial was discontinued after 102 patients had been randomised because of an insufficient rate of enrolment. Fifteen patients were excluded owing to absence or withdrawal of informed consent, leaving 87 patients for analysis: 48 in the loxapine group and 39 in the placebo group.

Except for age, the characteristics of the patients at inclusion were similar in the two groups, especially in terms of sedation dose in the 24 h before randomisation (Table 1).

**Table 1:** Baseline characteristics of the intention-to-treat population

	Loxapine (*n* = 48)	Placebo (*n* = 39)	*p* Value
Age, years (SD)	59.6 (15.6)	51 (17.7)	*0.02*
Male sex, *n* (%)	35 (74.5)	31 (79.5)	0.6
SAPS II score (SD)	48.2 (17.2)	53.7 (17.9)	0.15
SOFA score [IQR]	9 [5–12]	9 [7–11]	0.8
Alcohol consumption, *n* (%)	13 (28)	17 (44)	0.12
Toxic drug abuse, *n* (%)	5 (11)	6 (15)	0.54
Psychoactive drug use, *n* (%)	8 (17)	10 (26)	0.33
Indication for MV			
Acute respiratory failure,﻿ *n* (%)﻿	23 (49)	16 (41)	0.46
Shock, *n* (%)	12 (25)	9 (23)	0.48
Coma﻿, *n* (%)﻿	8 (17)	9 (23)	0.48
Co-morbid conditions			
COPD, *n* (%)	5 (11)	4 (10)	1.0
Congestive heart disease, *n* (%)	6 (13)	4 (10)	1.0
Chronic renal failure,* n* (%)	2 (4)	0 (0)	0.5
Hepatic disease, *n* (%)	1 (2)	1 (3)	1.0
Duration of MV before randomisation, days [IQR]	5 [3–7]	6 [5–8]	0.06
Cumulative amount of sedative drugs in the previous 24 h			
Midazolam, mg	114 [40–230]	120 [57–341]	0.5
Fentanyl, μg	2150 [1731–4000]	2700 [2550–4300]	0.6
Sufentanil, μg	247.5 [107–333.75]	270 [130–638.75]	0.2
Propofol, mg	1440 [750–2550]	1520 [1085–2400]	0.3

Abbreviations: *COPD* Chronic obstructive pulmonary disease, *MV* Mechanical ventilation, *SAPS II* Simplified Acute Physiology Score II (calculated in the first 24 h of admission in ICU), *SOFA* Sepsis-related Organ Failure assessment (calculated at randomisation)

Data are *n* (%), median (IQR) or mean (SD)

The median time from ICU admission to study inclusion did not differ significantly between the loxapine group (5.5 [3.49–7.78] days) and the placebo group (5.66 [4.49–6.81] days) (*p* = 0.47).

### Outcomes

#### Primary outcome

**Fig. 3:**
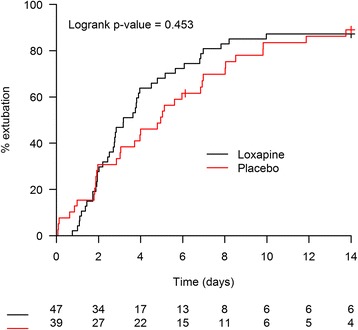
Kaplan-Meier plot. Proportion of successful extubation during the 14 days following randomisation

During the 14-day period from randomisation, the median times to successful extubation were 3.2 days in the loxapine group and 5 days in the placebo group (relative risk 1.2, 95% CI 0.75–1.88, *p* = 0.45). Figure 3 shows the daily rate of successful extubation in the loxapine and placebo groups until day 14. Three patients died within the 14 days of follow-up (one in the loxapine group, two in the placebo group), and only one death (placebo group) occurred when a patient was still under MV.

To investigate the intersecting appearance of the two curves, we performed a supplementary data analysis of the first 24 h after randomisation. This analysis revealed that six patients were extubated in the placebo group (including four unplanned extubations) and two were extubated in the loxapine group (planned extubations), accounting for a higher initial rate of extubation in the placebo group. The results of the primary outcome analysis after 14 days for a median follow-up of 29.5 days did not differ (data not shown).

#### Secondary outcome

**Fig. 4:**
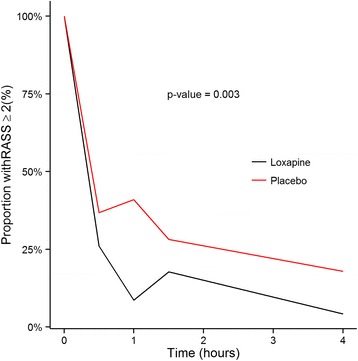
Proportion of patients with agitation (Richmond Agitation-Sedation Scale [RASS] score ≥ 2) during the first 4 h following randomisation. Sedation resumption may explain the RASS score reduction in placebo group. However, this reduction was more frequent in the loxapine group

Sedation was more frequently resumed in the placebo group (44% vs 17%, *p* = 0.01) during the first 24 h (Table 2), and the proportion of patients with RASS scores ≥ 2 was significantly lower in the loxapine group (*p* = 0.003) during the 4 h following study drug administration (Fig. 4).

**Table 2:** Secondary outcomes

	Loxapine	Placebo	*p* Value
Ventilator-free days in first 14 days (SD)	5.8 (8.4)	5.5 (8.4)	0.9
Unplanned extubations, *n* (%)	6 (13)	7 (18)	0.5
Sedation resumption during the first 24 h, *n* (%)	8 (17)	17 (44)	*0.01*
Nosocomial pneumonia, *n* (%)	7 (15)	8 (21)	0.5
Mortality at 14 days, *n* (%)	1 (2)	2 (5)	0.6
Mortality at 6 weeks, *n* (%)	5 (10)	3 (8)	0.7

We identified no difference in either ventilator-free days during the first 14 days or rate of unplanned extubation, nosocomial pneumonia or mortality after 14 days and 6 weeks (Table 2). Total dose administered within the first 24 h did not differ between groups (2 [2, 3] vs 3 [2–4], *p* = 0.22). However, the total dose administered within the first 48 h and within 14 days were higher in the placebo group (4 [2–5] vs 5 [4–6], *p* = 0.03; and 4 [3–7] vs 8 [4–14], *p* = 0.01).

**Fig. 5:**
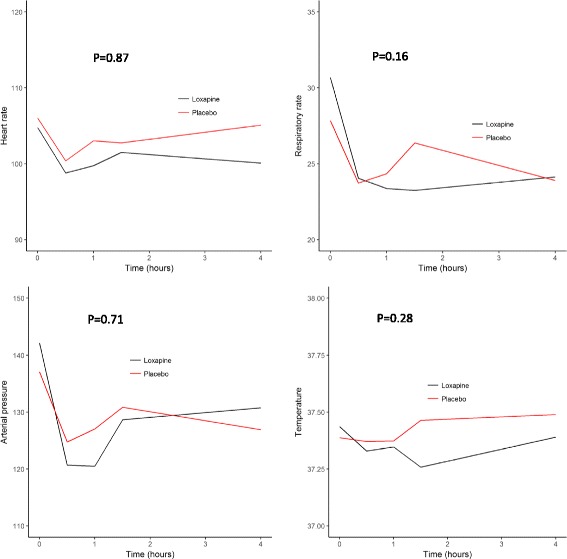
Evolution of physiological parameters (arterial pressure, heart rate, respiratory rate and temperature) during the first 4 h after randomisation

The evolution of physiological parameters (arterial pressure, heart rate, respiratory rate and temperature) was not different during the first 4 h after randomisation (Fig. 5). Owing to a substantial amount of missing data, P0.1 was not analysed. Univariate analysis did not identify any factor associated with unsuccessful weaning 5 days after randomisation, particularly with regard to the randomisation group.

The rate of adverse events did not differ between the two groups (Table 3). One patient in the loxapine group had a transient seizure episode.

**Table 3:** Main adverse events

	Loxapine	Placebo	*p* Value
Shock, *n* (%)	5 (10)	6 (15)	0.54
Unexplained fever, *n* (%)	1 (2)	0 (0)	1.0
Seizure, *n* (%)	1 (2)	0 (0)	1.0
Dyskinesia, *n* (%)	0	0	

## Discussion

In this prematurely stopped study of the effects of loxapine in agitated patients during weaning from invasive MV, loxapine did not significantly shorten weaning from MV. However, loxapine administration significantly reduced the RASS score in the 4 h after randomisation and also reduced the need for sedation resumption in the subsequent 24 h.

We hypothesised that loxapine might allow for a decrease of median duration of MV of 2 days. This hypothesis might have been correct because we observed median times from randomisation to successful extubation of 5 days in the placebo group and of 3.2 days in the loxapine group. Unfortunately, we randomised only 102 patients instead of the 300 initially scheduled, and analysis was possible in only 87 of them (because of absence or withdrawal of consent in 15), which precludes any conclusion based on the primary hypothesis. With the observed sample size, this study has a power of 40% to demonstrate a decrease of 2 days of median duration of MV in the loxapine group and a power of 80% to demonstrate a decrease of 3 days of median duration of MV in the loxapine group.

Lack of power might potentially explain the absence of difference in the primary outcome. Difficulties in recruiting patients were a consequence of the infrequency of isolated agitation during MV weaning, as attested by the flowchart. Several causes may explain these difficulties. First, use of sedative drugs has decreased in the ICU in recent years [15] as a probable consequence of major publications that promote less sedation [3, 16]. In our study, participating centres used a written sedation protocol or daily sedation interruption [16] that could lead to less profound sedation and therefore less agitation during awakening. Second, participating centres took great care in conducting pain evaluation and management, which may also have resulted in less frequent agitation occurrence. Third, the non-inclusion criterion of extubation planned in the following 24 h could be too subjective. Indeed, during the screening period, 19 patients were not included because of this criterion.

This study is the first double-blind, placebo-controlled, randomised trial in which loxapine for agitation was tested during weaning from invasive MV. We found that loxapine significantly reduced agitation, as attested by RASS score decrease in the first 4 h after randomisation, without significant adverse outcomes. Sedative drug administration (increase of dosage or resumption) is often required to control agitation during weaning from invasive MV [17]. In our study, sedation resumption was needed significantly less often with loxapine than with placebo. This result is an important strength of this study because prolonged sedation has several drawbacks, including limitation of clinicians’ ability to interpret physical examinations; promotion of delirium [18]; and prolongation of invasive MV, ICU stay and hospital stay [3, 19].

The intersecting appearance of the two curves shown in Fig. 2 is explained by more unplanned extubation in the placebo group during the 24 h following randomisation (four unplanned extubations in the placebo group vs none in the loxapine group). Unplanned extubation may be associated with serious complications [20]. Although the effects of unplanned extubation on nosocomial pneumonia are debated [21–23], unplanned extubation seems to prolong ICU stay [24], and it is an indicator of quality of care in the ICU [23, 25].

Our trial has potential limitations. First, only three centres used written protocols for sedation. However, a significant influence of sedation management on the primary outcome is unlikely because it is a double-blind, placebo-controlled trial. Second, the use of non-invasive ventilation to treat post-extubation respiratory failure may create heterogeneity in the primary endpoint. Unfortunately, data on non-invasive respiratory support (non-invasive ventilation, high-flow oxygen nasal cannula) were not collected. These heterogeneities in practice may constitute confounding factors. However, such confounding is very unlikely to affect the results. Indeed, if it had any effect, it would likely be to decrease the capacity of proving a difference (type II error). Our study was stopped prematurely after the inclusion of one-third of the expected population. This constitutes the most plausible explanation for lack of significance. The effect of confounding, if any, would be to further decrease the significance of the results. On the contrary, it seems more than improbable that confounders would falsely increase the probability of finding a significant difference (type I error) because our results are negative. In any case, a larger-scale randomised controlled trial is mandatory to evaluate the potential of loxapine in this context and should minimise heterogeneity, taking the above-mentioned confounding factors into account.

The dose of loxapine in the protocol followed the industry instruction and was validated by our hospital pharmacist. Antipsychotic agents have been tested more often to prevent or treat delirium in the ICU [6, 26–29] than for agitation during weaning from MV. A pilot randomised, placebo-controlled study including 36 patients showed that quetiapine added to as-needed haloperidol resulted in faster delirium resolution and less agitation [27]. No larger randomised trial was performed to confirm these results. More recently, in a randomised, double-blind, placebo-controlled trial including 142 patients, intravenous haloperidol did not modify the duration of delirium in critically ill patients [6]. In this trial, patients receiving haloperidol were less frequently agitated, however, leading the authors to conclude that the use of haloperidol should be reserved for short-term management of acute agitation. Haloperidol has numerous drawbacks, including extra-pyramidal manifestations and significant QTc interval prolongation [30, 31]. Because of its good haemodynamic safety profile and the rarity of its side effects, loxapine has been used routinely in many ICUs in France. We observed a single episode of seizure in one patient in the loxapine group, but no other adverse effects. This seems to confirm the safety of use of this drug, but it emphasises the need for close patient monitoring. We previously showed that loxapine allowed for adequate control of agitation with normalisation of several physiological parameters during weaning from MV [7]. To our knowledge, these potentially interesting properties have not previously been tested in a randomised controlled trial. In addition, there is a lack of data on the dose of loxapine which is required to control agitation in the ICU. Our study suggests that a first administration of 150 mg and then a maintenance dose of 50–100 mg every 4 or 8 h in case of persistent agitation is safe and efficient for treating acute agitation.

## Conclusions

Loxapine allowed agitation control during MV weaning and decreased the need for sedation resumption compared with placebo. However, loxapine did not significantly shorten weaning from MV. Altogether, our study constitutes a firm rationale for undertaking a more powered one to assess the potential benefit of loxapine in agitated patients during weaning from MV.

## Abbreviations

COPD: Chronic obstructive pulmonary disease
ICU: Intensive care unit
MV: Mechanical ventilation
RASS: Richmond Agitation-Sedation Scale
SAPS II: Simplified Acute Physiology Score II
SOFA: Sepsis-related Organ Failure assessment
